# Brain Networks Reveal the Effects of Antipsychotic Drugs on Schizophrenia Patients and Controls

**DOI:** 10.3389/fpsyt.2019.00611

**Published:** 2019-09-12

**Authors:** Emma K. Towlson, Petra E. Vértes, Ulrich Müller-Sedgwick, Sebastian E. Ahnert

**Affiliations:** ^1^Center for Complex Network Research and Department of Physics, Northeastern University, Boston, MA, United States; ^2^Media Laboratory, Massachusetts Institute of Technology, Cambridge, MA, United States; ^3^Department of Psychiatry, Behavioural and Clinical Neuroscience Institute, University of Cambridge, Cambridge, United Kingdom; ^4^Department of Psychiatry, University of Cambridge, Cambridge, United Kingdom; ^5^Barnet Enfield Haringey Mental Health NHS Trust, Springwell Centre, Barnet Hospital, London, United Kingdom; ^6^Theory of Condensed Matter Group, Department of Physics, Cavendish Laboratory, University of Cambridge, Cambridge, United Kingdom; ^7^Sainsbury Laboratory, University of Cambridge, Cambridge, United Kingdom

**Keywords:** schizophrenia, fMRI, network science, brain network, antipscychotic medication

## Abstract

The study of brain networks, including those derived from functional neuroimaging data, attracts a broad interest and represents a rapidly growing interdisciplinary field. Comparing networks of healthy volunteers with those of patients can potentially offer new, quantitative diagnostic methods and a framework for better understanding brain and mind disorders. We explore resting state functional Magnetic Resonance Imaging (fMRI) data through network measures. We construct networks representing 15 healthy individuals and 12 schizophrenia patients (males and females), all of whom are administered three drug treatments: i) a placebo; and two antipsychotic medications ii) aripiprazole and iii) sulpiride. We compare these resting state networks to a performance at an “N-back” working memory task. We demonstrate that not only is there a distinctive network architecture in the healthy brain that is disrupted in schizophrenia but also that both networks respond to antipsychotic medication. We first reproduce the established finding that brain networks of schizophrenia patients exhibit increased efficiency and reduced clustering compared with controls. Our data then reveal that the antipsychotic medications mitigate this effect, shifting the metrics toward those observed in healthy volunteers, with a marked difference in efficacy between the two drugs. Additionally, we find that aripiprazole considerably alters the network statistics of healthy controls. Examining the “N-back” working memory task, we establish that aripiprazole also adversely affects their performance. This suggests that changes to macroscopic brain network architecture result in measurable behavioral differences. This is one of the first studies to directly compare different medications using a whole-brain graph theoretical analysis with accompanying behavioral data. The small sample size is an inherent limitation and means a degree of caution is warranted in interpreting the findings. Our results lay the groundwork for an objective methodology with which to calculate and compare the efficacy of different treatments of mind and brain disorders.

## Introduction

In recent years, neuroimaging data and graph theory have allowed for the description of the topological properties of large-scale brain networks (1–3). Disorders of the brain have long been thought to be due to abnormal connectivity patterns, and these networks allow for a quantitative measure of this disruption (4, 5). Schizophrenia is a debilitating psychiatric condition with a range of symptoms, including auditory and visual hallucinations, delusions, disorganized thinking, and cognitive impairment. Various network-based studies have associated schizophrenia with a subtle randomization of connections (6–11). Antipsychotic medications are employed to treat symptoms with varying degrees of success and side effect. Sulpiride is a selective dopamine antagonist most commonly used in Europe and Japan for schizophrenia treatment. Aripiprazole is an atypical third-generation antipsychotic introduced for the treatment of schizophrenia in the USA in 2002 and Europe in 2004 (12). It acts as a dopamine receptor partial agonist, whereas typical antipsychotics used to combat the symptoms of schizophrenia are pure dopamine antagonists. Partial agonists have long been of interest (13) to avoid the extrapyramidal and endocrine side effects caused by typical antipsychotics.

There is a body of evidence demonstrating drug treatments lead to specific localized changes in functional network structure (14, 15), and a growing interest in whole-brain approaches (16). Yet, few graph theoretical studies to date have been conducted to understand if and how medication alters an individual’s whole-brain network (17–19). We hypothesized that an effective medication would act to make the functional brain networks of patients more similar to those of healthy volunteers. We set out to test this in the context of three drug treatments: i) placebo; ii) aripiprazole, and iii) sulpiride. We used resting state fMRI data to analyze the functional connectivity, and a working memory task to assess the cognitive abilities, of 15 healthy volunteers and 12 patients with chronic schizophrenia.

Our results show that schizophrenia patients and healthy controls exhibit different network topologies, in agreement with the existing literature (7, 10, 20). Further, the antipsychotic drug treatments alter the topology of the brain network in a measurable way, particularly in healthy individuals. In the brain networks of patients, we found evidence that the antipsychotic drugs lead to network topologies that are closer to those of healthy individuals. This correlates with improved cognitive performance. In healthy individuals, treatment with aripiprazole leads to a significantly altered network, as well as lower cognitive performance.

## Materials and Methods

### Experimental Design and Statistical Analysis

#### Sample

Twelve people with chronic schizophrenia and 15 healthy, nonpsychotic volunteers were recruited for participation in this study (see **Supplementary Datasheet 2** for detailed demographics). The patients were diagnosed according to standard operational criteria in the DSM-IV (21) and were clinically stable during their involvement (i.e., exhibiting low symptom ratings and undergoing no change of medication in the preceding 4 weeks). All were receiving antipsychotic drugs, and four were receiving additional psychotropic medication, but were not treated with their usual medication on the days of scanning to avoid effects on the fMRI data. Healthy volunteers were selected to match the patient group in terms of age, gender, premorbid IQ, years of education, and handedness, and screened for major psychiatric disorders using the Mini International Neuropsychiatric Interview (22). All subjects provided informed consent in writing, and the protocol was approved by the Addenbrooke’s NHS Trust Local Research Ethics Committee.

Every subject attended three scanning sessions, each 1 to 2 weeks apart, for collection of functional MRI data and completion of working memory tests (see below). At each visit, they were administered one of three drug treatments: i) an oral placebo, 180 and 90 min before scanning; ii) oral aripiprazole, 15 mg 180 min before scanning and oral placebo, 90 min before; iii) oral placebo, 180 min before scanning and oral sulpiride, 400 mg 90 min before. We used a double dummy design with dosing of aripiprazole 180 min and sulpiride 90 min before the start of fMRI scanning. Both patients and experimenters were blind for the drug condition. The study medication was randomized by a colleague, who was not a member of the study team and stored in envelopes for each patient and testing session. There was a sealed envelope with the drug order for each participant that could be opened in case of a serious adverse effect. Both aripiprazole and sulpiride are antipsychotic medications designed to alleviate the symptoms of schizophrenia. At both time points (−180 min and −90 min), we co-administered 10 mg of domperidone to minimize side effects. Domperidone is a peripheral D_2_ receptor blocker sometimes used to mitigate nausea in pharmacological functional neuroimaging studies (23, 24). It does not cross the blood-brain barrier and will therefore not confound the neuroimaging results. The order of drug administration and the playlists of the working memory paradigm were counterbalanced across one group and repeated for the other.

#### Working Memory Tests

At each session, the subjects were required to complete an “N-back” task to assess their verbal working memory (25–27). The task demanded that subjects maintain a series of visually presented letters in their working memory such that each stimulus could be compared with the letter presented N letters earlier in the series (i.e., N-back)—see **Figure 2A**. For example, if the sequence of letters was F-B-A-B, the subject could be expected to indicate on presentation of the last letter in the series that B was presented two letters previously (2-back). Difficulty was manipulated to four levels (0-back to 3-back) by varying the number of letters back in the series that the subject had to compare to the current letter. All subjects were provided with written instruction and completed a practice version before undertaking the full task. They completed three playlists matched for difficulty and distraction, which were allocated across sessions such that each subject would complete each playlist once across their three visits (for placebo, aripiprazole, and sulpiride). An individual’s performance at this cognitive task was assessed by recording their hit rate, defined as the proportion of times they were able to successfully present a correct answer. In each session, there were 10 correct targets for each N-back level. Data for the performance of patients 11 and 12 administered sulpiride were missing so analyses were carried out without them, leaving *n* = 10 patients and *n* = 15 healthy subjects.

#### Acquisition and Preprocessing of fMRI Data

A General Electric (GE) Signa system scanner operating at 1.5 T at the BUPA Lea Hospital (Cambridge, UK) was used to acquire functional MRI data over 17 min and 12 s, during which time, subjects were asked to lie quietly with their eyes closed. In each session, 516 gradient-echo T2*-weighted echo planar images depicting blood oxygenation level-dependent (BOLD) contrast were generated from 16 noncontiguous near-axial planes: repetition time, 2 s; echo time, 40 ms; flip angle, 70°; voxel size, 3.05 × 3.05 × 7.00 mm; section skip, 0.7 mm; matrix size, 64 × 64; field of view (FOV), 240 × 240 × 123 mm. Four volumes were discarded to allow for T1 equilibration effects, leaving 512 volumes per data set (10).

Control 2 was missing an anatomical image so was discarded from the study, and patient 11 was missing data for the aripiprazole treatment. Each data set was analyzed for effects of head motion within the scanner (28–30), resulting in the further rejection of patient 3 on aripiprazole and sulpiride, control 10 on placebo and sulpiride, control 8 on sulpiride, and patient 5 on placebo, all of which were deemed to have too many motion-related artefacts to be reliable (see **Supplementary Presentation 1**). The remaining data sets were corrected for motion through realignment and wavelet despiking (31, 32). We used a 12-parameter affine transformation to register the data to MNI stereotactic standard space and a 6-mm Gaussian kernal for spatial smoothing. Finally, the voxel time series were high- and low-pass filtered with cutoff frequencies of ≈ 0.01 Hz and ≈ 0.08 Hz, respectively, as per (28, 31). Statistical analyses of subsequent network metrics were performed using those data sets which were available for all drug treatments, equating to 9 patients and 12 controls.

#### Kolmogorov-Smirnov Test

The two-sample *Kolmogorov-Smirnov test* is a non-parametric test to compare two sets of data. The Kolmogorov-Smirnov statistic is a measure of the distance between the empirical distribution functions of the two samples and is calculated under the null hypothesis that both samples are taken from the same continuous probability distribution. The statistic can then be used to assign a p-value to the likelihood that the null hypothesis may be rejected. We used this method to assess the distribution of the global network measures for each of the six groups: controls (×3—aripiprazole, placebo, sulpiride) and patients (×3—aripiprazole, placebo, sulpiride). A p-value < 0.05 was taken to indicate a significant result.

#### Analysis of Variance (ANOVA)

To examine the effects of the drugs, volunteer type, and task difficulty on cognitive performance, we performed a three-way ANOVA with two repeated measures (33) in various combinations on the hit rates of the subjects. A p-value < 0.05 was taken to indicate a significant result.

#### Cohen’s *d*

To quantify effect sizes between groups, we calculated Cohen’s *d* (34), which is defined as:

(1) $$d=\frac{{\bar{x}}_{1}-{\bar{x}}_{2}}{\sigma_{\mathrm{pooled}}}$$

where ${\bar{x}}_{1}$ and ${\bar{x}}_{2}$ are the means of the two samples and $\sigma_{\mathrm{pooled}}=\sqrt{\frac{(n_{1}-1)\sigma_{1}^{2}+(n_{2}-1)\sigma_{2}^{2}}{n_{1}+n_{2}-2}}$ is the pooled standard deviation for the two samples of size *n*_1_ and *n*_2_.

#### Games-Howell *Post Hoc* Test

The Games-Howell *post hoc* test is a modification of the traditional Tukey’s Honestly Significant Differences (HSD) test for data sets with unequal variances and/or sample sizes. For two groups with means ${\bar{x}}_{1}$ and ${\bar{x}}_{2}$ and standard deviations σ_1_ and σ_2_, the critical *q* *_crit_* can be looked up in the Studentized *t* statistic *q* table with a modified degrees of freedom:

(2) $$df^{\prime }=\frac{{\left(\frac{\sigma_{i}^{2}}{n_{i}}+\frac{\sigma_{j}^{2}}{n_{j}}\right)}^{2}}{\frac{{\left(\frac{\sigma_{i}^{2}}{n_{i}}\right)}^{2}}{n_{1}-1}+\frac{{\left(\frac{\sigma_{j}^{2}}{n_{j}}\right)}^{2}}{n_{j}-1}}$$

Then, we consider the difference between the means, which, to be considered significant, must satisfy:

(3) $${\bar{x}}_{1}-{\bar{x}}_{2}\geq q_{\mathrm{crit}}\sqrt{\frac{\frac{\sigma_{i}^{2}}{n_{i}}+\frac{\sigma_{j}^{2}}{n_{j}}}{2}}$$

We employ the Games-Howell *post hoc* test to examine the drug treatment effects on the patients alone, and use a significant level of 0.05.

### Anatomical Parcellation and Wavelet Decomposition

For each individual data set, voxel time series were averaged within each of the 325 equally sized anatomical regions in a random driven atlas [see Ref. (35) for approach]. Twenty-eight regions lacked good-quality fMRI time series for some subjects, so were discarded from our analysis, leaving data sets for 297 brain regions for all subjects. The discarded regions are mostly cerebellar, an area known to be highly susceptible to artefacts due to the major arteries which pass in the vicinity (36), and the complete list can be found in the **Supplementary Presentation 1**. The maximal overlap discrete wavelet transform (37) was used to decompose each individual regional mean fMRI time series into the frequency interval 0.030 to 0.060 Hz (scale 3). This frequency range was selected as it is has been shown that frequencies ≤ 0.1 Hz exhibit the most prominent salient neuronal fMRI dynamics (38).

### Topological Network Construction

Undirected weighted networks were generated for each individual based on correlating scale 3 wavelet coefficients. The resulting correlation coefficients *r* *_ij_* form the weight of the edges connecting regions *i* and *j*. A simple thresholding procedure was then applied to eliminate edges with weights smaller than τ; all remaining edges are then given a weight of 1, providing an undirected, unweighted network. The threshold τ can be varied to generate networks with any desired percentage of possible connections. Following the example of Lynall et al. (10), which studies a subset of this particular data set, we choose to focus on 37% to 50% connectivity. First, this ensures that all graphs are connected, and second, it avoids the increasing randomness associated with higher connection densities (39). All results given for the unweighted networks are averages across this range.

### Global Network Measures

#### Clustering

**Figure 1:**
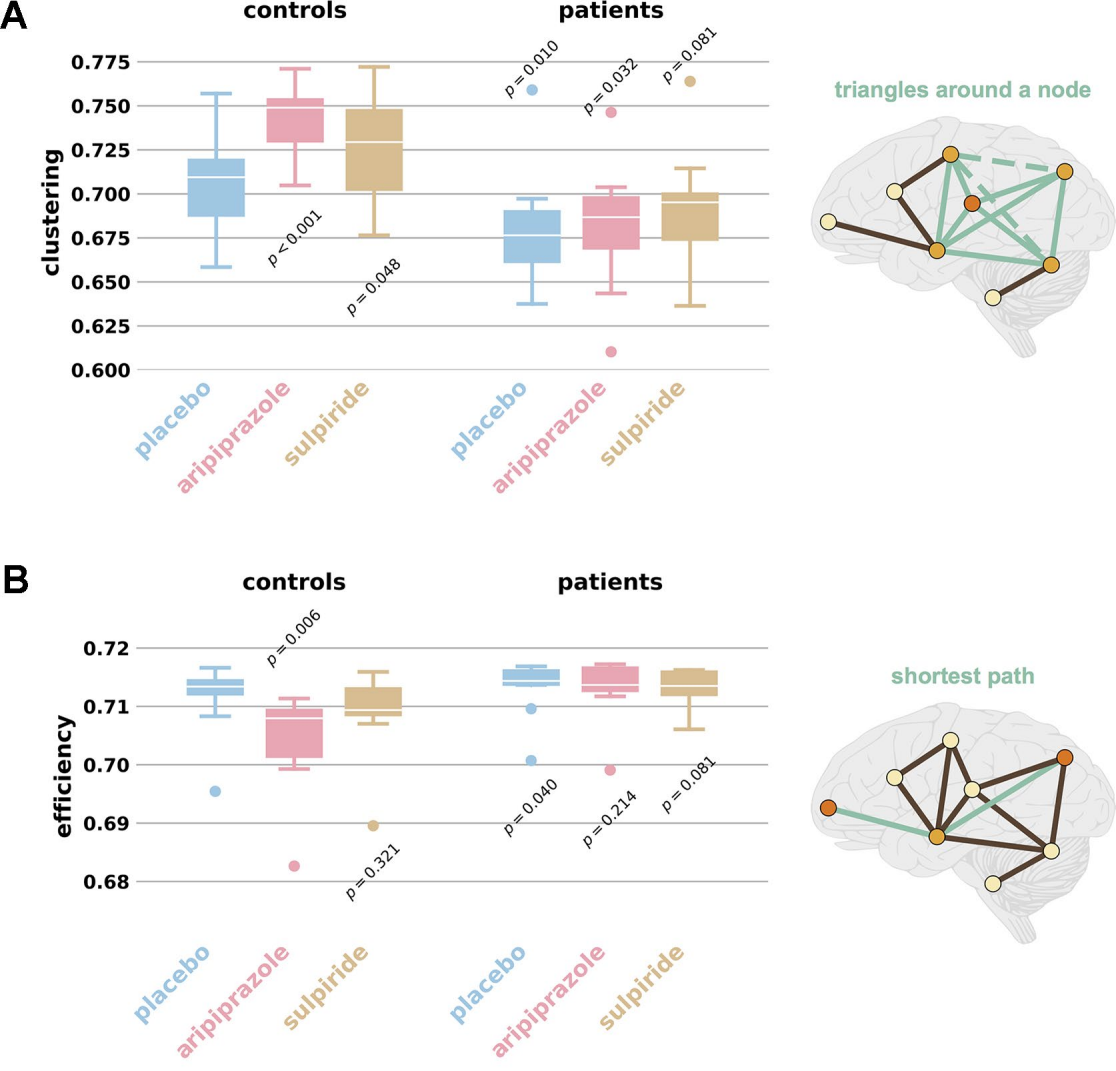
Average clustering and global efficiency values. The box plots display distributions of **(A)** average clustering and **(B)** global efficiency for the networks of each group and drug, for individuals with networks for every drug treatment. The extreme ends of the whiskers correspond to the maxima and minima and the white line in the box corresponds to the median. Controls (*n* = 12) are grouped on the left and patients (*n* = 9) on the right. Placebo is shown in blue, aripiprazole in pink, and sulpiride in gold. *p* values refer to likelihood the distributions match that of the control placebo group (two-sample K-S test). Outliers are defined as being more than 1.5× the interquartile range away from the median; note this is purely visual and no values are excluded from statistical analyses. Schizophrenia is associated with lower clustering and higher efficiency, seen by comparing the control placebo plot to the patient placebo plot. The antipsychotic medications increase clustering and decrease efficiency, therefore moving patients closer to controls and affecting the control networks. All values can be found in **Supplementary Datasheet 1** and the demographics of all participants can be found in **Supplementary Datasheet 2**. The schematics illustrate the concepts of **(A)** clustering and **(B)** efficiency. **(A)** Clustering measures the number of triangles which exist around a node (green solid lines), as a proportion of those that could (also green dashed lines). **(B)** Efficiency averages the inverse shortest paths (green lines) between all node pairs; many short paths equates to higher efficiencies.

The *clustering coefficient*, *C* *_i_*, for a node *i* can be defined as (40):

(4) $$C_{i}=\frac{2T(i)}{k_{i}(k_{i}-1)}$$

where *T*(*i*) is the number of triangles containing node *i*, and *k* *_i_* is the degree of *i*—see **Figure 1A**.

The *average clustering* then provides a global network measure of clustering, and is simply the average of all values of nodal clustering, or:

(5) $$C=\frac{1}{N}\sum_{i\in G}C_{i}$$

#### Characteristic Path Length

If the shortest path lengths, *L* *_ij_*, between all existing node pairs *i* and *j*, are identified, then the *characteristic path length* of the network, *L*, is simply given by the mean of their sum:

(6) $$L=\frac{1}{N(N-1)}\sum_{i\neq j\in G}L_{\mathrm{ij}}$$

#### Efficiency

A measure of the *global efficiency* of a network, *E* *_Global_*, is given by the mean of the sum of the inverse shortest path lengths, *L* *_ij_*, between all existing node pairs *i* and *j* (17, 41):

(7) $$E_{\mathrm{Global}}=\frac{1}{N(N-1)}\sum_{i\neq j\in G}\frac{1}{L_{\mathrm{ij}}}$$

where *N* is the number of nodes in the graph *G*. Networks for which the average path length is small can thus be said to have high global efficiency (17) (see **Figure 1B**).

This is equivalent to averaging the nodal efficiencies for all nodes in the network.

#### Assortativity

The *assortativity* of a network is a measure of the preference of its nodes to connect to other nodes of similar degree. Let *e* *_xy_* be the joint probability distribution (or mixing matrix) of the degrees. Then if $\sum_{y}e_{\mathrm{xy}}=a_{x}$ and $\sum_{y}e_{\mathrm{xy}}=b_{y}$ are, respectively, the fraction of edges that start and end at vertices with values *x* and *y* and further that *e* *_xy_* ≠ *a* *_x_* *b* *_y_* (the case of no assortative mixing), the assortativity coefficient can be defined simply by calculating the Pearson correlation coefficient (42):

(8) $$A=\frac{\sum_{\mathrm{xy}}\mathrm{xy}(e_{\mathrm{xy}}-a_{x}b_{y})}{\sigma_{a}\sigma_{b}}$$

where σ*_a_* and σ*_b_* are the standard deviations of the distributions *a* *_x_* and *b* *_y_*. *A* has a value in the range −1 ≤ *r* ≤ 1, where *A* = 1 would correspond to a perfect correlation between *x* and *y*, i.e., perfect assortativity, and similarly, *A* = −1 would indicate perfect disassortativity.

### Software

Motion diagnostics, preprocessing and parcellation of the functional MRI data were completed using the preprocessing pipeline with temporal despiking from (31, 43). Metric calculations and network manipulations were carried out using the Python networkx library (44) and Matlab. We used IBM SPSS Statistics for all ANOVA calculations (45).

## Results

### The Effects of Schizophrenia on Global Efficiency and Clustering Are Mitigated by Medication

We first compared the functional brain networks derived from schizophrenia patients and healthy volunteers on placebo treatments and, as expected (7, 10), find distinct differences. In agreement with the existing literature, the schizophrenia networks have moderately increased efficiency (median *E* *_Global_*_,_ *_SZ_* = 0.7144 compared with *E* *_Global_*_,_ *_HV_* = 0.7132, *p* = 0.04, *d* = 0.38) and a large reduction in clustering (median *C* *_SZ_* = 0.6765 compared with *C* *_HV_* = 0.7079, *p* = 0.01, *d* = 1.06)—see **Figure 1**. With clustering and efficiency values intermediate between those of random graphs and lattices, both the healthy and patient networks exhibit small-world properties (46).

We next employed an ANOVA (two-way, one repeated-measure) to examine any differences in network measures between groups, and the factors underlying them. This confirmed group differences due to subject type on both efficiency and clustering (*p* = 0.010 and *p* = 0.002, respectively) and also indicated group differences due to drug effect (*p* = 0.041 and *p* = 0.05 respectively)—see **Tables 1** and **2**, and **Supplementary Datasheet 1**. Our hypothesis was that the antipsychotic medications would aim to make the brain connectivities of patients more similar to those of healthy individuals. In the light of the observed differences between the control and patient placebo groups, this hypothesis translates to an expectation that aripiprazole and sulpiride will reduce efficiency and increase clustering. We do indeed find this for the healthy volunteers, but only small changes for the patients (**Figure 1**). Moreover, after each antipsychotic drug treatment, the brain networks of people with schizophrenia had global efficiencies which were no longer statistically different from the healthy brain networks (median *E* *_Global_* = 0.7137, *p* = 0.241, *d* = 0.16 for aripiprazole and *E* *_Global_* = 0.7135, *p* = 0.081, *d* = 0.37 for sulpiride). Both antipsychotic drugs also led to average clustering coefficients that were much closer to those of healthy brain networks (median *C* = 0.6867, *p* = 0.032, d = 0.83 for aripiprazole, *C* = 0.6952, *p* = 0.081, *d* = 0.60 for sulpiride). Post hoc tests revealed that differences in efficiency and clustering across drug treatments in patients alone are not significant (see **Supplementary Datasheet 4**).

**Table 1:** Summary statistics for a 2 way ANOVA with 1 repeated measure on the network global clustering values of patients and healthy controls treated with placebo, aripiprazole, and sulpiride. Individuals for which networks were available for all drug treatments were used, equating to n = 12 for healthy controls and n = 9 for patients. There is a significant difference between the network clustering of the HV and SZ groups (p = 0.002), a significant drug effect (p = 0.005) and an additional drug-group type interaction term (p = 0.005)—all highlighted in red. This interaction term stems from the effect of aripiprazole—it greatly increases the clustering of control networks while causing only a small and variable increase in the schizophrenia networks. The placebo and sulpiride treatments have a more consistent effect on the two groups.

Source	SS	df	MS	F	p
**Between groups**					
Subject type	<0.001	1	<0.001	8.093	0.010
Error	0.001	19	<0.001		
**Within groups**					
Drug	<0.001	2	<0.001	3.480	0.041
Drug×Subject type	<0.001	2	0.0001	1.005	0.376
Error	0.0010	38	<0.001		

**Table 2:** Summary statistics for a two-way ANOVA with one repeated measure on the network global efficiency values of patients and healthy controls treated with placebo, aripiprazole, and sulpiride. Individuals for which networks were available for all drug treatments were used, equating to *n* = 12 for healthy controls and *n* = 9 for patients. There is a significant difference between the network efficiency of the HV and SZ groups (*p* = 0.010) and a significant drug effect (*p* = 0.041)—highlighted in red. Individuals for which networks were available for all drug treatments were used, equating to *n* = 12 for healthy controls and *n* = 9 for patients. There is a significant difference between the network efficiency of the HV and SZ groups (*p* = 0.010) and a significant drug effect (*p* = 0.041).

Source	SS	df	MS	F	p
**Between groups**					
Subject type	0.026	1	0.026	12.208	0.002
Error	0.040	19	0.002		
**Within groups**					
Drug	0.004	2	0.002	6.023	0.005
Drug^x^Subject type	0.004	2	0.002	5.997	0.005
Error	0.012	38	<0.001		

### Aripiprazole Significantly Changes Healthy Brain Networks

We found that aripiprazole has a dramatic effect on healthy individuals, with a large variation across individuals. We observe significantly reduced global efficiencies (median *E* *_Global_* = 0.7080, σ = 0.009, *p* = 0.006, *d* = 1.08) and an even greater increase in clustering (median *C* = 0.7490, *p* < 0.001, *d* = 1.63). Sulpiride increased clustering in healthy networks (median *C* = 0.7294, *p* = 0.028, *d* = 0.74), but had no significant effect on global efficiency (median *E* *_Global_* = 0.7094, *p* = 0.321, *d* = 0.43). Almost all metrics examined are greatly altered in the healthy volunteers administered with aripiprazole, indicating considerable restructuring of functional connectivity (see **Figure 1** and **Supplementary Datasheet 1**). These results are consistent with observations in the brain networks of people with schizophrenia, the drug treatments reduce efficiency and increase clustering.

### Cognitive Performance of Healthy Individuals is Impaired After Taking Aripiprazole

**Figure 2:**
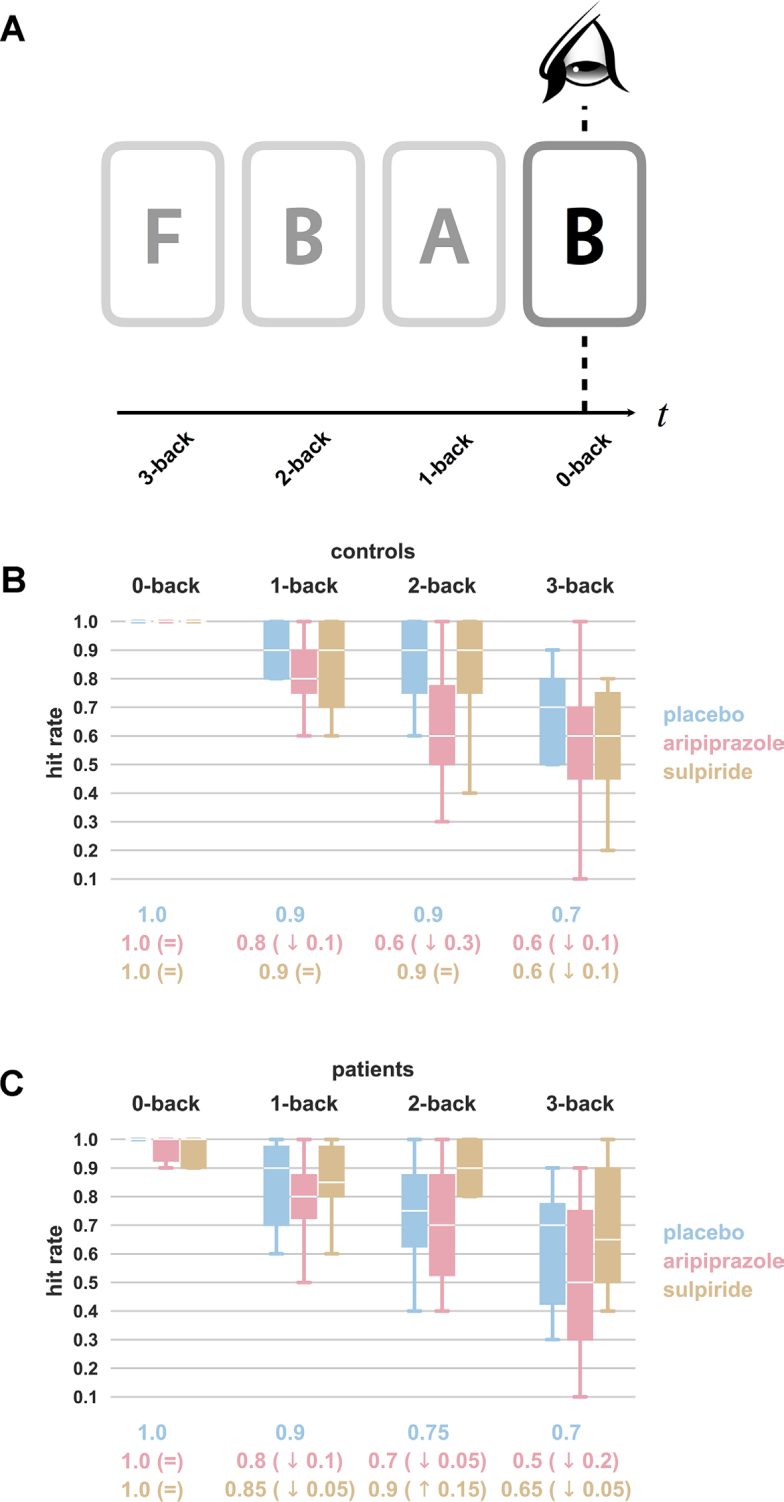
N-back working memory task. Panel **(A)** illustrates the nature of the task. Subjects are shown a sequence of letters, and asked to indicate if they are presented with a letter which matches the letter presented “N-back.” In the example shown, the subjects are expected to note for the 2-back task that B was presented two letters previously (and for the 0-back task that they are observing the current letter). There is nothing to note for the 1-back and 3-back tasks. Naturally, 0-back is the easiest version of the task and 3-back the hardest. For each drug treatment, hit rates (or fraction of correct responses out of a total of 10 prompts) from **(B)** healthy individuals (*n* = 15) and **(C)** schizophrenia patients (*n* = 10) averaged across each level of difficulty are presented as box plots. Results for placebo are shown in blue, aripiprazole in pink, and sulpiride in gold. The extreme ends of the whiskers correspond to the maxima and minima, and the white line in the box corresponds to the median. Values below the boxes represent the median values, and for the drug treatment groups, the difference with the median of the placebo group is provided in brackets. Aripiprazole is associated with a reduced number of correct answers as compared with placebo, most strikingly so for controls completing the 2-back task. All values can be found in **Supplementary Datasheet 3**.

All subjects score consistently highly on the very easy 0-back task, but their performance deteriorates considerably with increasing difficulty of the task, with subjects experiencing profound difficulty with the 3-back version (see **Figure 2**, **Table 3**, and **Supplementary Datasheet 3**). In the healthy cohort, aripiprazole has a detrimental effect on performance, whereas the impact of sulpiride is negligible—see **Figure 2B**. In the challenging 2-back version of the task, the disparity is most clear—aripiprazole has a negative impact on cognitive performance, giving rise to an average hit rate of 0.65 ± 0.21 (compared with 0.83 ± 0.19 on placebo). Sulpiride, however, has no noticeable impact, with subjects achieving an average hit rate of 0.83 ± 0.18.

**Table 3:** Summary statistics for a three-way ANOVA with two repeated measures on the hit rates during a working memory task with four levels of difficulty of patients and healthy controls treated with placebo, aripiprazole, and sulpiride. Individuals for which data were available for all drug treatments were used, equating to *n* = 15 for healthy controls and *n* = 10 for patients. We see a significant effect of cognitive difficulty (*p* < 0.001) and a significant drug effect (*p* = 0.007)—highlighted in red.

Source	SS	df	MS	F	p
**Between groups**					
Subject type	0.007	1	0.007	0.044	0.836
Error	3.762	23	0.164		
Within groups					
Drug	0.386	2	0.193	5.489	0.007
Drug^x^Subject type	0.011	2	0.006	0.160	0.853
Error	1.618	46	0.035		
Cog. difficulty	4.828	3	1.609	42.779	<0.0001
Cog. difficulty^x^Subject type	0.045	3	0.015	0.403	0.751
Error	2.596	69	0.038		
Drug^x^Cog. difficulty	0.120	6	0.020	1.160	0.331
Subject type^x^Drug^x^Cog. difficulty	0.162	6	0.027	1.160	0.331
Error	2.384	138	0.017		

An ANOVA (three-way, two repeated measures) revealed that, naturally, the predominant factor in determining success at the working memory tests was the difficulty of the task (*p* < 0.0001) (see **Table 3**). However, it also demonstrated that the drug treatments have a significant effect (*p* = 0.007). We then separated out the drug treatments into placebo-aripiprazole and placebo-sulpiride groups and repeated the ANOVA on controls and patients separately. This shows that the effect is only observed in the healthy volunteers, and that aripiprazole is the medication responsible (p = 0.015, *d* = 0.37 for the placebo-aripiprazole groups and *p* = 0.769, *d* = 0.04 for the placebo-sulpiride groups). Thus, among healthy volunteers, we find that aripiprazole results in poorer performance at the N-back working memory task as compared with placebo, and that sulpiride has no noticeable effect.

### Cognitive Tests Show Worse Performance of Patients but do not Capture Drug Effects

Patients scored worse than controls in the N-back working memory task, but not significantly so, with an average hit rate of 0.73 ± 0.18 for patients and 0.83 ± 0.19 for controls for the placebo 2-back task. Aripiprazole and sulpiride do not change the performance of patients much (average hit rates of 0.71 ± 0.21 and 0.79 ± 0.28, respectively, for the 2-back task) with an ANOVA showing no significant drug effect (*p* = 0.217) (see **Figure 2C**).

## Discussion

### Network Topology, Illness, and Medication

It has been known for some time that the whole-brain functional brain network organization of people with schizophrenia differs from that of healthy volunteers (7, 10). However, very few graph-theoretic studies have been conducted into the effect of antipsychotic medication, or indeed any drug for any brain disorder, on this network organization. The ones that have been conducted found measurable drug effects (17, 18, 47, 48) [including converse to treatment, inducing psychosis (49)]. We hypothesized that a drug designed to treat schizophrenia would modify the brain connectivities of patients, making them more similar to those of healthy individuals. The results for global efficiency and clustering in **Figure 1** clearly demonstrate this principle—sulpiride and aripiprazole act to reduce efficiency in both controls and patients. While the differences among the patient group do not prove to be significant, they do leave the patients with network efficiencies and clustering comparable to those of the unmedicated controls, and the controls have lower efficiency and higher clustering than before. In addition, we also observed a strong effect of a single dose of aripiprazole on medication-naive healthy controls. This finding is consistent with our result in patients, as the drug seemingly tries to “correct” for schizophrenia network characteristics—in the absence of schizophrenia—by altering the network metrics to decrease efficiency and increase clustering.

### Network Topology and Cognitive Ability

Our results suggest that there is an optimal configuration for a brain network in terms of maximizing cognitive ability: performance worsens given any change (increase *or* decrease) in the examined metrics from a healthy baseline, the control placebo group. We saw that as well as having a characteristically different brain network structure, schizophrenia patients perform less well at tests of cognitive ability than their healthy counterparts, as has been previously demonstrated (4), although these effects are not significant. Further, we showed that the group who performed most differently on medication (healthy volunteers having been administered aripiprazole) was also the group who had the most changes to the topology of their brain networks. This supports the notion that one’s cognitive ability is intrinsically linked to the structure of the brain’s functional network (50). For example, to integrate and process information quickly, a network requires some level of efficiency. The control group on aripiprazole had diminished efficiency and performed significantly worse at the N-back tasks than when on placebo. No such impaired performance is seen for sulpiride, for which the reduction in efficiency was negligible. On the other hand, the schizophrenia brain networks appear to have *too* high an efficiency, perhaps leading to disordered or overwhelming information integration, and they too perform worse. The drugs do non-significantly decrease efficiency in patients, and their performance is slightly improved at the 1- and 2-back tasks. A previous study on MEG-derived networks using the N-back working memory paradigm (51) demonstrated a shift toward a more random network configuration (with a decrease in modularity and clustering, and an increase in global efficiency) as the cognitive demands of the task increased. The authors also note significant differences from purely random networks and argue that global sychronization is important in higher cognition, which is reflected in the network architecture.

### Neurochemical Differences in Schizophrenia

Schizophrenia implicates D_2_ receptors (among others) (52, 53), and a succession of studies have demonstrated increased presynaptic dopamine synthesis in psychosis and at-risk patients (54, 55). Dopamine antagonists, such as aripiprazole and sulpiride, are therefore used to treat schizophrenia. PET studies of schizophrenia patients have found that greater dopamine receptor occupancy by aripiprazole was associated with better working-memory performance in terms of error rate and reaction time (56). Conversely, in healthy volunteers, greater striatal D_2_ receptor occupancy by aripiprazole was related to greater decrease in frontal metabolism, and greater reduction in frontal metabolism was associated with impaired performance at a working memory task (57). Thus, striatal dopaminergic function could contribute to the working-memory impairments observed in schizophrenia, and antipsychotic drugs could mitigate this by reducing excess striatal dopaminergic neurotransmission. With no excess dopamine synthesis in healthy volunteers, a reduction induced by dopamine antagonists leads to worsened performance.

Consistent with the literature, our results indicated that aripiprazole significantly worsened the performance of healthy subjects at the N-back working memory task, but did not hinder the patients. Sulpiride had no significant detected effects. The main pharmacological difference between the two antipsychotics is that aripiprazole is a partial D_2_ antagonist and sulpiride is the most selective D_2_ antagonist. Given this, we might expect that sulpiride have a larger detrimental effect on the healthy controls’ performance, but it is possible that due to the relatively high dose of aripiprazole, it had stronger D_2_ antagonistic effects due to the relatively high dose of sulpiride. It is also surprising that we observed such small differences between the healthy and patient groups at the working memory tasks. However, we recruited relatively stable and high-functioning patients, and all participants were trained outside the scanner so that their performance was relatively stable. We aimed to match patients and controls for task performance so that drug effects were not confounded by group effects at the baseline (the placebo treatment).

### Limitations

The greatest limitation of this study is the small sample size: *n* = 15 for healthy controls and *n* = 12 for patients with schizophrenia, which is further reduced to *n* = 12 and *n* = 9, respectively, for the functional brain networks, and *n* = 15 and n = 10, respectively, for the working memory tasks. The fMRI time series were collected with standard parameters at the time of study, which given the age of the data sets, inevitably have some drawbacks compared with modern state-of-the-art data sets. Most notably, this includes a greater sparsity of imaging, compounded by the loss of 28 regions due to head motion artefacts. However, we do note that the acquisition time was long (17 min 12 s), which will have benefited the accuracy of the regional correlations and, therefore, also, the networks we derived from them (58). There is a considerable literature comprising studies based on this type of data (10, 46), and importantly, key network-based results have been replicated in more modern studies (19, 59).

Finally, there are likely confounding effects from other drugs. All patients were prescribed oral antipsychotic medication and asked not to take their medication on the days of the fMRI study (see **Supplementary Datasheet 2** for the details of their usual medications). While this mitigates acute pharmacological effects, patients had been treated with these other antipsychotics for several years, and their long-term effects cannot be accounted for. This limitation affects the analysis and interpretation of group effects (differences between patient and controls), but not for the comparison of drug conditions within each group (of patients and controls). An earlier study utilizing a subset of these data (10) found no significant correlation with antipsychotic dosage (in chlorpromazine equivalents) and any of the connectivity or network metrics they examined.

### Brain Networks as a Means to Assess Medication

This quite unique data set allowed for an investigation into the effects of antipsychotic drugs on both the large-scale functional brain networks and the cognitive performance of people diagnosed with chronic schizophrenia and healthy volunteers. Despite its limitations, clear drug effects were observed on the network topology and performance at the N-back working memory task. We find a reduction in the difference between specific healthy and patient network metrics and that aripiprazole impairs cognitive ability and radically rewires the brain networks of healthy volunteers. It would be highly beneficial for future studies to use state-of-the-art functional MRI data to further investigate the links between disrupted networks in people with brain disorders, how medication influences these, and an “ideal” network topology for the brain (which should be identified by association with an optimal behavioral parameter, such as the best cognitive performance). Such advanced studies have the potential for not just diagnosis of the original brain disorder, but also for the quantification of the effectiveness of a drug in treating the illness. This would then allow for a systematic comparison between alternative treatments.

## Data Availability

The datasets for this manuscript are not publicly available because the fMRI data were collected with formal approval from the Addenbrooke’s NHS Trust Local Research Ethics Committee in 2005. They did not approve making anonymized data available. Requests to access the datasets should be directed to ET (ektowlson@gmail.com).

## Ethics Statement

This study was carried out in accordance with the recommendations of the Addenbrooke’s NHS Trust Local Research Ethics Committee with written informed consent from all subjects. All subjects gave written informed consent in accordance with the Declaration of Helsinki. The protocol was approved by the Addenbrooke’s NHS Trust Local Research Ethics Committee. All participants were provided with a detailed PIS that explained the nature of the pharmacological experiment and the double dummy design.

## Author Contributions

ET and SA conceived of the analysis. UM-S designed the clinical trials, recruited the subjects, and gathered the data. ET did the analysis, SA oversaw it, and ET, SA, and PV interpreted the results. ET, PV, and SA wrote the manuscript.

## Funding

ET was supported by an Engineering & Physical Sciences Research Council (UK) PhD studentship, and is now supported by NSF award number 1734821. PV was supported by the Medical Research Council (MR/K020706/1), and is now a Fellow of MQ: Transforming Mental Health, grant number MQF17_24. The Behavioural & Clinical Neuroscience Institute is supported by the Medical Research Council (UK) and the Wellcome Trust. SA was supported by a Royal Society University Research Fellowship (UF080037 and UF120247), and is currently supported by a Gatsby Career Development Fellowship (PTAG/021). Data collection was supported by a grant from Bristol Myers Squibb to the late Robert Kerwin at King’s College London.

## Conflict of Interest Statement

UM-S has received honoraria for advisory board participation, consultancy, and educational talks, travel expenses, and/or support for educational conferences from Astra Zeneca, Bristol Myers Squibb, Eli Lily, Heptares, Lundbeck, Flynn Pharma/Medice, Janssen-Cilag, Shire, and/or Sunovion.

The remaining authors declare that the research was conducted in the absence of any commercial or financial relationships that could be construed as a potential conflict of interest.
