# Supplementary material for: Brain Networks Reveal the Effects of Antipsychotic Drugs on Schizophrenia Patients and Controls
Source: Front Psychiatry. 2019 Sep 12;10:611. doi: 10.3389/fpsyt.2019.00611 (PMC6752631; doi:10.3389/fpsyt.2019.00611)
Supplement: Supplementary file 5 [file Presentation_1.pdf]

Brain networks reveal the effects of antipsychotic drugs on schizophrenia patients and controls: Supplementary information

Preprocessing and correcting for head motion

We completed a number of preprocessing steps on the raw fMRI data. The skull was removed from the images and the data deobliqued. The 16 slices which comprised a volume were gathered alternately and we temporally corrected them by phase shifting the signal. We used a 12 parameter affine transformation to register the data to MNI stereotactic standard space and a 6mm Gaussian kernal for spatial smoothing. Finally, the voxel timeseries were high- and low-pass filtered with cutoff frequencies of ≈ 0.01 Hz and ≈ 0.08 Hz respectively. As described in the main text, for each individual dataset voxel time series were averaged within each of the 325 equally sized anatomical regions in a random driven atlas (Zalesky et al., 2010). 28 regions lacked good quality fMRI timeseries for some subjects so were discarded from our analysis, leaving datasets for 297 brain regions for all subjects. The 28 regions we discarded, largely in the cerebellum, are:

Right Superior Temporal Gyrus

Left Inferior Frontal Gyrus

Left Cerebellar Tonsil

Right Pyramis

Left Inferior Frontal Gyrus

Right Cerebellar Tonsil

Left Parahippocampal Gyrus

Right Cerebellar Tonsil

Right Culmen

Right Uvula

Left Cerebellar Tonsil

Left Inferior Semi-Lunar Lobule

Left Precentral Gyrus

Left Cerebellar Tonsil

Right Superior Temporal Gyrus

Left Superior Temporal Gyrus

Right Cerebellar Tonsil

Right Culmen

Right Cerebellar Tonsil

Right Fusiform Gyrus

Left Cerebellar Tonsil

Right Cerebellar Tonsil

Left Cerebellar Tonsil

Right Cerebellar Tonsil

Left Culmen

Right Inferior Frontal Gyrus

Right Cerebellar Tonsil

Right Cerebellar Tonsil

Whilst in the scanner, subjects invariably move; even the act of swallowing leads to undesirable head motion effects. In addition to the obvious problem of physically displacing the brain, motion introduces a spin history effect. Very precise spatial and temporal alignment of magnetic gradients is required to acquire the BOLD signal. Protons which move from one voxel to another have different excitations than expected and cause intensity changes in the BOLD measurements. For each volume, the position of the head must be estimated and realigned to a reference position (typically the middle volume). The realignment is done via a rigid body transform described by 6 parameters (x, y and z translations, and pitch, yaw and roll rotations) that is estimated to minimise the registration error. To attempt to remove spin history effects,

measures such as ICA decomposition and regression of the motion parameters are typically undertaken.

Three independent studies were published in 2012 Power et al. (2012); Satterthwaite et al. (2012); Van Dijk et al. (2012) demonstrating that these measures were insufficient, that motion artefacts remained in the data despite them, and that they introduced systematic errors in regional correlations. Scans of patients and young participants were found to be especially problematic. Frames can be identified as contaminated by motion effects by examining the framewise displacement (a measure of how much the head moved between frames) and DVARS (a measure of brain-wide rate of change of BOLD intensity in one frame) Power et al. (2012). We used the same cutoffs as Power et al. (2012) to determine unacceptable amounts of motion: values greater than 0.5 for framewise displacement and 0.5% ΔBOLD for DVARS, with the additional requirement that at least 5 mins worth of data should be deemed of acceptable quality for a subject to remain in the study. It was proposed that one should *scrub* the data, which simply equates to removing any bad frames. This is undesirable as it usually results in the loss of a large proportion of data and produces discontinuous timeseries. An alternative recently developed approach uses wavelet despiking Patel et el (2014). Head motion is often sporadic and leads to stochastic spikes in the data which are difficult to identify. A wavelet transform decomposes the timeseries into different frequency scales within which such spikes are more apparent.

Each of our datasets was analysed for effects of head motion within the scanner as described, which resulted in the rejection of patient 3 on aripiprazole and sulpiride, control 10 on placebo and sulpiride, control 8 on sulpiride and patient 5 on placebo, all of which were deemed to have too many bad frames to be reliable. The remaining datasets were corrected for motion through realignment and wavelet despiking Patel et el (2014); Suckling et al. (2006). Following the example of Power et al. (2012), Figure 1 contains plots of the Euclidean distance between regions against the average of Δr for each cohort, where Δr is the difference between regional correlations in scrubbed and unscrubbed versions of the data. We observe no relation between

the two, indicating that scrubbing has no systematic effect and we have successfully removed unwanted motion-related signals.

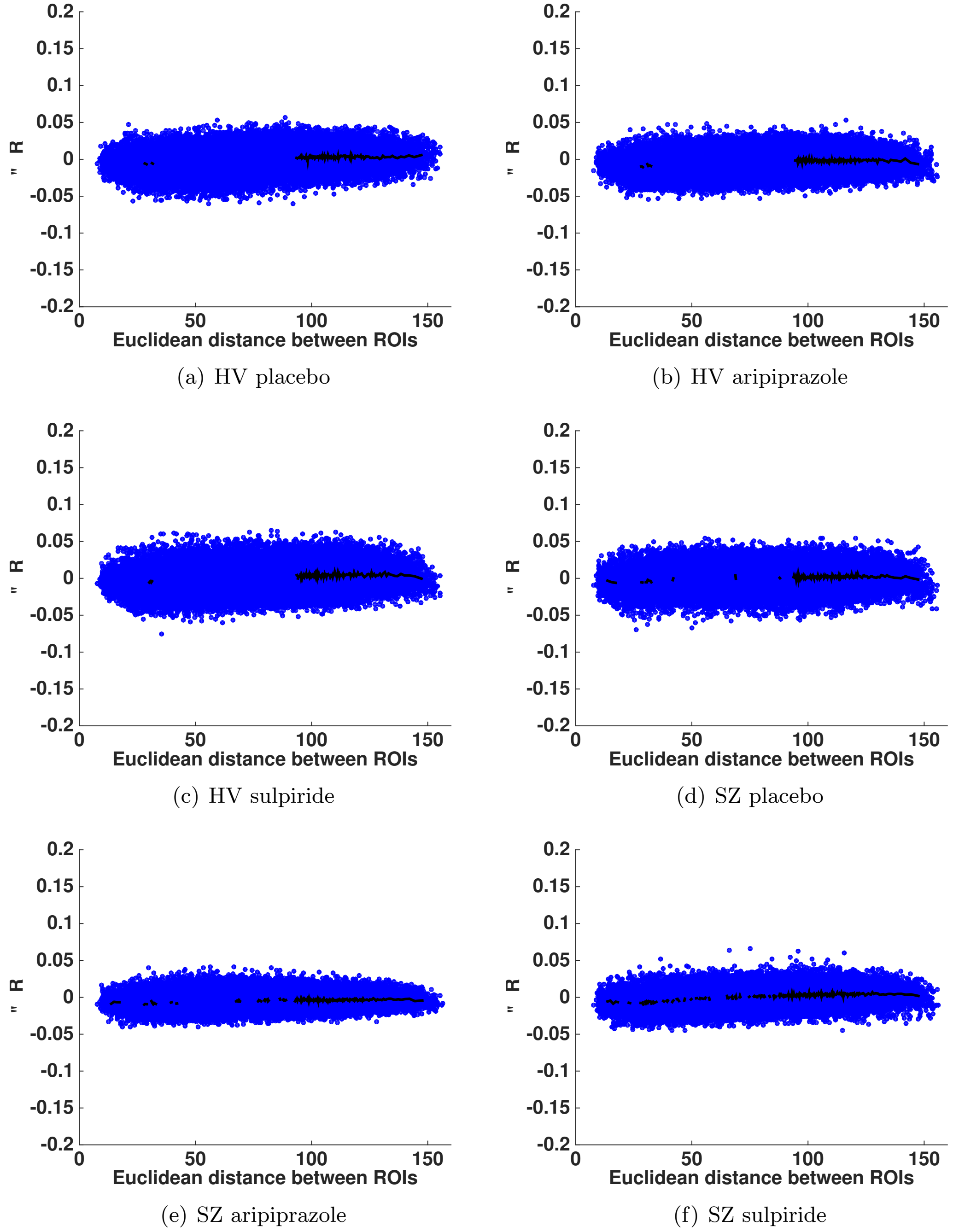

Figure 1. Cloud plots to determine the presence of motion effects. Plots of Euclidean distance between regions against the average Δr for each cohort (Δr the difference between scrubbed and unscrubbed derived regional correlations). No correlation is observed indicating no significant motion effects.

References

Patel AX, Kundu P, Rubinov M, Jones PS, Vértes PE, Ersche KD, Suckling J and Bullmore (2014) A wavelet method for modeling and despiking motion artifacts from resting-state fMRI time series. *Neuroimage* 95:287-304.

Power JD, Barnes KA, Snyder AZ, Schlaggar BL and Petersen SE (2012) Spurious but systematic correlations in functional connectivity MRI networks arise from subject motion. *Neuroimage* 59(3):2142-54.

Satterthwaite TD, Wolf DH, Loughead J, Ruparel K, Elliott MA, Hakonarson H, Gur RC and Gur RE (2012) Impact of in-scanner head motion on multiple measures of functional connectivity: relevance for studies of neurodevelopment in youth. *Neuroimage* 60(1):623-32.

Suckling J, Long C, Triantafyllou C, Brammer M, Bullmore ET (2006) Variable precision registration via wavelets: Optimal spatial scales for inter-subject registration of functional MRI. *Neuroimage* 31(1):197-208

Van Dijk KR, Sabuncu MR and Buckner RL (2012) The influence of head motion on intrinsic functional connectivity MRI. *Neuroimage* 59(1):431-8.

Zalesky A, Fornito A, Harding IH, Cocchi L, Yöcel M, Pantellis C and Bullmore ET (2010) Whole-brain anatomical networks: does the choice of nodes matter? *Neuroimage* 50(3):(970-83).
